# Multi-Group Regularized Gaussian Variational Estimation: Fast Detection of DIF

**DOI:** 10.1017/psy.2024.15

**Published:** 2025-01-03

**Authors:** Weicong Lyu, Chun Wang, Gongjun Xu

**Affiliations:** 1 Faculty of Education, University of Macau, Macau, China; 2 College of Education, University of Washington, Seattle, WA, USA; 3 Department of Statistics, University of Michigan, Ann Arbor, MI, USA.

**Keywords:** differential item functioning, latent variable modeling, regularization, variational estimation

## Abstract

Data harmonization is an emerging approach to strategically combining data from multiple independent studies, enabling addressing new research questions that are not answerable by a single contributing study. A fundamental psychometric challenge for data harmonization is to create commensurate measures for the constructs of interest across studies. In this study, we focus on a regularized explanatory multidimensional item response theory model (re-MIRT) for establishing measurement equivalence across instruments and studies, where regularization enables the detection of items that violate measurement invariance, also known as differential item functioning (DIF). Because the MIRT model is computationally demanding, we leverage the recently developed Gaussian Variational Expectation–Maximization (GVEM) algorithm to speed up the computation. In particular, the GVEM algorithm is extended to a more complicated and improved multi-group version with categorical covariates and Lasso penalty for re-MIRT, namely, the importance weighted GVEM with one additional maximization step (IW-GVEMM). This study aims to provide empirical evidence to support feasible uses of IW-GVEMM for re-MIRT DIF detection, providing a useful tool for integrative data analysis. Our results show that IW-GVEMM accurately estimates the model, detects DIF items, and finds a more reasonable number of DIF items in a real world dataset. The proposed method has been integrated into R package VEMIRT (https://map-lab-uw.github.io/VEMIRT).

## 1 Introduction

Addressing broad scope research questions, such as the impact of medical, behavioral, and psycho-social interventions, is typically beyond the scope of a single research project and requires data from multiple studies to build a more cumulative science. Integrative data analysis (IDA) is a novel framework for conducting simultaneous analysis of raw data pooled from different studies. It offers many advantages, including increased power due to larger sample sizes, enhanced external validity and generalizability due to greater heterogeneity in demographic and psycho-social characteristics, cost-effectiveness due to the reuse of extant data, and potential to address new research questions not feasible by a single study, among others (Curran et al., 2010; Curran & Hussong, 2009). However, significant methodological challenges must be addressed when pooling data from independent studies, and one such challenge is to establish commensurate measures for the constructs of interest (e.g., Nance et al., 2017). When data from different yet overlapping instruments and diverse samples are pooled, the assumption of measurement invariance, often required by existing methods, would likely be violated.

Procedures for evaluating and establishing measurement equivalence across samples are well developed from both factor analysis and item response theory frameworks. These traditional methods focus on comparing independent groups defined by a single categorical covariate to determine if any items display differential item functioning (DIF, also known as item-level measurement non-invariance). More recently, Bauer (2017) proposed the moderated nonlinear factor analysis (MNLFA), a unified flexible model that can handle different types of study-specific covariates simultaneously, such as gender (categorical) and age (continuous), and can handle different types of responses. The cost of this generalization is the drastically increased model complexity that prohibits the adoption of conventional DIF detection methods simply because the resulting number of potential model comparisons would be huge. To overcome this problem, Bauer et al. (2020) proposed a regularized MNLFA by using a penalized likelihood function that imposes a Lasso (i.e., least absolute shrinkage and selection operator) penalty on DIF parameters. This procedure obviates the reliance on statistical hypothesis testing for DIF, but instead, the penalty term shrinks small DIF parameters directly toward zero, indicating that DIF is not detected on these item parameters. Although the Lasso penalty has been proven to have good performance under some conditions (van de Geer, 2008; Zhao & Yu, 2006), the theoretical guarantee of Lasso (such as oracle property) in item response theory models has yet to be established.

The current regularized MNLFA is only restricted to unidimensional constructs, while this work aims to expand the methodology to accommodate multidimensional constructs. This is an important step forward as many theoretical constructs in behavioral and health measurement in general are related, complex, and multifaceted (Fayers, 2007; Michel et al., 2018; Zheng et al., 2013). For instance, HIV stigma, a barrier to HIV testing and counseling, status disclosure, partner notification, and antiretroviral theory (ART) access and adherence, is found to have at least two dimensions: emotional stigma and physical stigma (Carrasco et al., 2017). In addition, clinical patient-reported outcome measures (PROMs) have been increasingly endorsed, or even mandated by policymakers and payers as a means of gauging not only a treatment’s benefits, but also its appropriateness. Since multi-trait assessment has emerged as a fundamental requirement for patient-centered decision making, the methodology also needs to advance on par with the demand. From a statistical perspective, using a multivariate approach would also produce more accurate factor scores with reduced standard errors of measurement by borrowing information from correlated scales.

In this study, we focus on a regularized explanatory multidimensional IRT (re-MIRT) model that handles potential item measurement non-invariance (i.e., DIF), thereby adjusting for, for instance, between-study heterogeneity. With proper penalty such as Lasso, fitting re-MIRT on the integrated data will output a commensurate scale for multidimensional constructs (e.g., depression, anxiety, alcohol use) that well accounts for study-specific idiosyncrasy resulting from the diversity of study populations and the use of different instruments. In addition, for the common items shared among studies, re-MIRT automatically tests for measurement invariance and corrects for non-invariance when spotted. Hence, the final factor scores from re-MIRT are cleaned from the contamination of DIF and they can be readily used in subsequent statistical analyses for addressing critical research questions.

In recent literature, Wang et al. (2023) first used the Lasso-type penalty with the two-dimensional two-parameter logistic model, and their proposed methods outperform the likelihood ratio test approach, especially when the proportion of DIF items is high. However, the regularization method can be slow because it requires a full estimation for each candidate tuning parameter value. When a large grid of tuning parameters is considered, the entire algorithm may take hours to finish. In this study, we aim to overcome these difficulties by leveraging the recently developed Gaussian Variational Expectation-Maximization (GVEM) algorithm (Cho et al., 2021) for MIRT models, which relies on a variational lower bound to approximate the true marginal likelihood, to speed up the computation. We generalize the GVEM algorithm to the more complicated DIF analysis setting with categorical covariates. To obtain a tighter lower bound for more accurate DIF detection, we further incorporate the importance sampling approach as an additional step after GVEM estimation to reduce the estimation bias (Ma et al., 2023). Compared to existing DIF detection methods, our proposed method is more efficient and scalable to higher dimensions and large-scale data, while still performing well in DIF detection. In addition, the source code for the proposed method is made available in R package VEMIRT, which can be accessed at https://map-lab-uw.github.io/VEMIRT.

The rest of the article is organized as follows. We first introduce the re-MIRT model for binary responses, followed by the regularized GVEM algorithm and bias reduction methods. Then we present two simulation studies and a real data analysis to evaluate the performance of the proposed algorithm. This article ends with some final discussions.

## 2 Method

### 2.1 Regularized explanatory MIRT

Let *N*, *J*, *K*, and *G* denote the numbers of persons, items, latent dimensions, and groups, respectively. For a dichotomously scored item *j*, the probability that person *i* with a latent trait vector 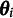 gives a correct response to item *j* is modeled as (1)

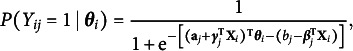

where 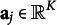 is a vector of discrimination parameters of item *j*, 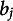 is a difficulty parameter of item *j*, and 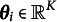 is a vector of latent traits for person *i*. The explanatory feature of the model is reflected by the inclusion of person level covariates, 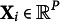 , which includes all the grouping information related to DIF (Wilson et al., 2008). In this study, we focus on a simpler case where person level covariates uniquely determine the group membership, i.e., 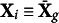 for all 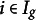 where 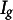 is the set of all persons in group *g*. 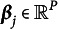 is a vector of regression coefficients implying the effect of grouping variables on the probability of correct response on item *j*. Similarly, 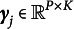 is a matrix of regression coefficients denoting the interaction effects of 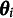 and grouping variables on item responses. By this way of parameterization, 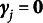 and 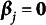 if item *j* does not have DIF, while 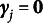 and 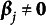 if item *j* has uniform DIF. Similar to the multiple-group IRT approach, the distribution of 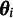 is allowed to differ across groups, i.e., 
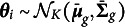
 for all 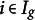 , which is known as impact.

Let 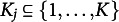 denote the set of latent dimensions that item *j* loads on, 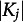 denote the cardinality of the set, and define 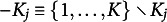 . For any 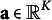 , 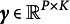 and 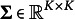 , let 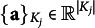 , 
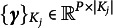
 and 
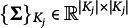
 be the slices of 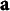 , 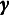 and 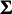 that keep the rows and/or columns indicated by 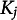 . As explained in Wang et al. (2023), in a confirmatory MIRT model, if 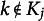 , then 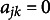 and 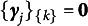 , i.e., the *k*th column of 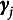 is a zero vector. Hence for each item *j*, we have 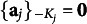 and 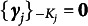 .

Denoting all model parameters by 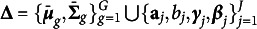 and the latent traits of all persons by 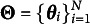 , the marginal likelihood of all the responses is 

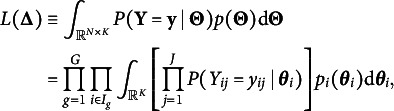

where 

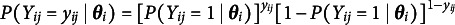

is the conditional likelihood, (2)

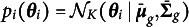

is the *K*-dimensional Gaussian density of 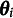 , and 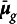 and 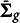 are the corresponding group-level population mean and covariance matrix, respectively.

Since persons *i* and *j* are in the same group if and only if 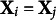 , we only need to consider the case where each 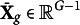 consists of 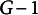 dummy variables indicating the group membership, that is, 

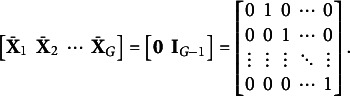

For groups 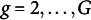 let 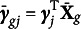 and 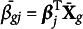 denote the DIF slope and intercept parameters of group *g* against group 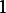 on item *j*, respectively. As the reference group, fix 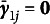 and 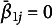 for 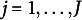 . Since 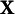 and 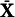 only contain group-level dummy variables, estimating 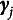 and 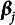 is equivalent to estimating 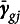 and 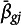 . To simplify notations, we let 
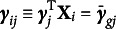
 and 
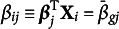
 for 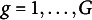 and 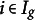 . Throughout this article we will gradually add more parameters to the model, and for simplicity we always let 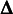 denote the current set of all the parameters to be estimated.

The “regularized” feature of the model is reflected by the Lasso or 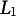 -penalized marginal log-likelihood function (3)

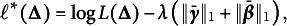

where (4)

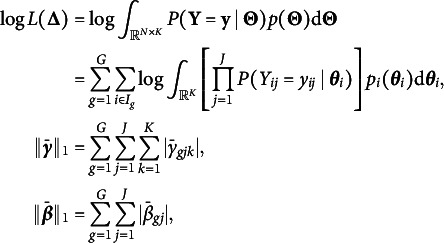

and 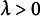 is a prespecified regularization parameter that controls sparsity (Wang et al., 2023). We will discuss a data-driven method which selects 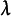 using information criteria in Section 0.2.4. Since (4) involves *K*-dimensional integrals which are intractable when *K* is large, directly maximizing (3) is challenging and approximation methods are needed.

### 2.2 Regularized multi-group GVEM

#### 2.2.1 Variational estimation

We generalize the Gaussian variational EM algorithm for MIRT models in Cho et al. (2021) to the more complex multiple-group scenario. Variational approximation methods are emerging approaches in modern statistics and machine learning for large-scale data analysis (Blei et al., 2017). The primary idea of GVEM is to approximate the original marginal likelihood that involves intractable integrals with a computationally feasible form known as the variational lower bound.

Traditional EM algorithms require finding the posterior distributions of latent variables, 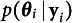 for each person *i*, by Bayes’ theorem within the E-step, which is intractable for large *K* due to the high-dimensional integral needed for computing marginal distributions. Variational EM algorithms, by contrast, approximate this unknown posterior distribution 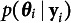 by a variational distribution 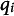 whose density is 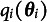 . Then the logarithm of the integral in (4) can be written as (5)

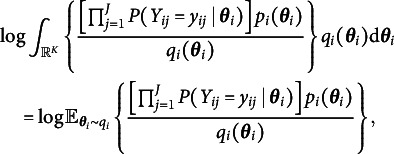

where the expectation in (5) is taken with respect to the variational distribution. By Jensen’s inequality, we obtain the evidence lower bound (ELBO) of (5) by switching the order of logarithm and expectation, i.e., (6)

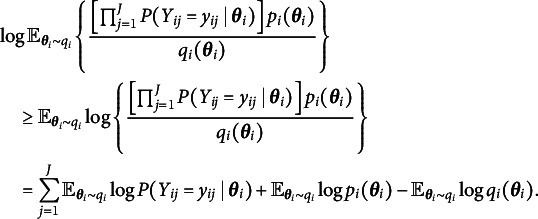

By carefully choosing the family of distributions where 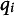 is from, we hope all terms in (6) have analytical solutions such that numerical integration is not necessary. Then, we estimate parameters by maximizing the ELBO instead of the intractable marginal log-likelihood function. The performance of this strategy depends heavily on how tight this lower bound is. Actually, it can be shown that the equality holds if and only if the Kullback–Leibler (KL) divergence 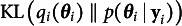 is zero, or equivalently 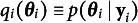 . Therefore, the key of the GVEM algorithm is to find a suitable 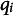 which not only approximates the posterior distribution well so that the KL divergence above is small but also leads to an ELBO that is easy to maximize. Following Cho et al. (2021), we choose 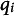 from the *K*-dimensional Gaussian family 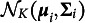 with 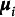 and 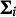 the mean vector and the covariance matrix of the Gaussian variational distribution respectively, and intend to maximize a variational lower bound of ELBO, (7)

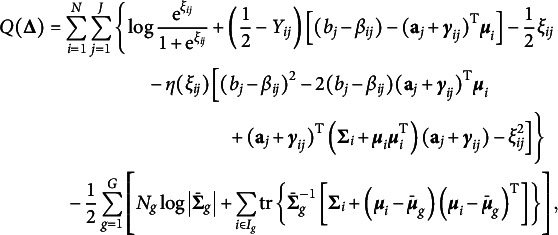

where 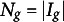 is the size of group *g*, 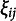 is a local variational parameter that helps simplify the estimation procedure (Cho et al., 2021), and 

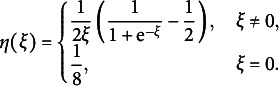

In the E-step we maximize (7) with respect to each variational distribution 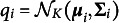 , which is equivalent to minimize 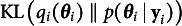 so that 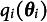 is the best approximation of 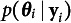 within the Gaussian family. This is different from the E-step in traditional EM algorithms where we let 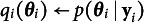 be the true posterior distribution such that 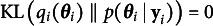 , which is ideal but leads to difficulty in computation. In the M-step we maximize (7) with respect to model parameters, including 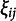 , 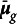 , 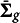 , 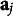 , 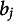 , 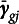 and 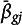 . In summary, the E-step and the M-step of GVEM are both “maximization” steps, but with respect to variational parameters and model parameters, respectively. Hence Rijmen and Jeon (2013) referred to it as the Maximization–Maximization (MM) algorithm. For GVEM, the two steps can be combined into one joint maximization step with respect to all the parameters in 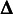 .

Maximizing (7) is straightforward for all the parameters except 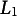 -penalized 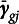 and 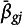 because we end up with a closed form updating formula for each parameter by letting the partial derivative of 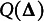 with respect to it be zero. There are no closed form updating formulas for 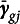 and 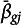 , so we adopt a quadratic approximation approach similar to Wang et al. (2023): the closed form update rule for entry 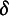 with respect to objective function *f* is 

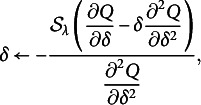

where 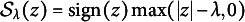 is a soft thresholding operator (Donoho & Johnstone, 1995). The updating formulas derived for all the parameters are shown in the Supplementary Material.

#### 2.2.2 Model identification

We need to fix 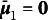 and 
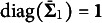
 for model identification. Here the subscript “ 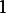 ” denotes the reference group, and users are free to define any group as the reference. However, the model is still not identified even with these two constraints when impact is present because any group 
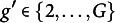
 can be rescaled without affecting other groups. Fixing any 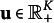 and 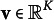 , for all 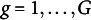 and 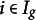 , we have the equality 

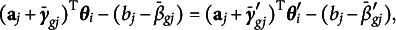

where 

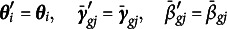

when 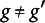 , and 

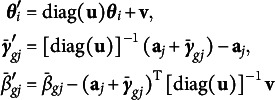

when 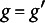 . For example, under uniform DIF such that 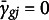 , consider the unidimensional case 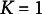 where 

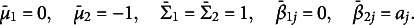

For any 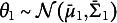 from group 1, consider corresponding 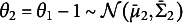 from group 2. Since 

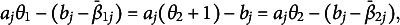

we cannot statistically distinguish between group 1 and group 2: group 2 has a lower mean 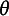 level, but its DIF in the intercept offsets this difference. However, although the two groups are statistically equivalent, group 1 does not have DIF since it is the reference group, while group 2 has DIF in the intercepts. Intuitively, all the items are more difficult to group 2 than to group 1 (i.e., 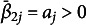 for all *j*) is equivalent to that group 2 has a lower mean latent trait level (i.e., 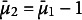 ).

Note that the possibility that DIF in item parameters is absorbed into differences in the distributions of latent traits across groups (i.e., impact) is not a problem if we have prior information about which items are DIF-free and hence can work as anchor items; any group differences detected on these items are attributed to impact rather than DIF (Chen et al., 2014). Even without such information, identifiability is still not an issue if we can safely assume that the proportion of DIF items is not too high. Since the regularization method penalizes non-zero DIF parameters, it favors sparse models with fewer DIF items and automatically lets non-DIF items be the anchors (Chen et al., 2023; Wang et al., 2023). The only difficult case is when there are too many DIF items (e.g., DIF proportion exceeds 50%). In the simulation study below we will consider the case where 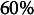 of the items have DIF. To distinguish between DIF and impact under such a challenging scenario, we generate balanced DIF effects (Debelak & Strobl, 2019), that is, there are both positive and negative DIF parameters that cancel out on average. Our proposed method turns out to work well on detecting such balanced DIF effects. Only if DIF occurs uniformly in one direction and DIF prevalence is higher than 50% that the method will not work well. It is worth emphasizing that the identification constraints are required by the model implied by (1) and (2), not the estimation algorithm. Regularization methods are already an improvement over other approaches that require pre-specified DIF-free items because they automatically look for them and set them as anchors.

#### 2.2.3 Debiasing lasso

After the EM algorithm converges, DIF parameters are determined because they have not been shrunk to exactly zero. No DIF is detected in item *j* if all entries in 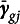 and 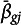 have been shrunk to zero for every group *g*, while any non-zero entry in 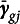 or 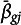 indicates DIF in item *j*. However, although DIF items have been determined, Lasso penalty is known to result in biased estimators for non-zero entries (Hastie et al., 2015). To better estimate model parameters and conduct model comparison for finding the best tuning parameter 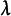 , it is necessary to re-estimate all the non-zero entries in 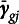 and 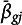 . Following Wang et al. (2023), debiasing can be done by running the EM algorithm again without a penalty (i.e., 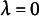 ) while fixing current zero entries in 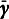 ’s and 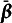 ’s at zero. Our final regularized GVEM algorithm for DIF detection is as follows:

1. Set initial values: 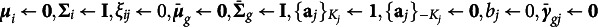 and 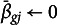 .
2. Repeat until convergence with 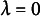 to find better initial values: in each iteration, update all the parameters using the closed form formulas.
3. For each 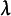 , start from the initial values obtained from Steps 1 and 2:
  - Repeat until convergence with the 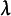 given: in each iteration, update all the parameters using the closed form formulas.
  - Repeat until convergence with 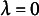 and current zero entries of 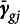 and 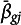 fixed at zero: in each iteration, update all the parameters using the closed form formulas.

Note that zero entries in 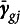 and 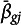 are determined by Step 3, and Step 3 is for debiasing non-zero entries only. The choice of initial values in Step 1 is arbitrary here, and researchers are encouraged to choose initial values based on their prior information. Step 2 is not necessary but helps speed up Step 3 by starting from better initial values.

### 2.3 Bias reduction via importance sampling

The multi-group GVEM algorithm is known to have a large bias in discrimination parameters, especially when the latent traits of different dimensions are highly correlated and the sample size is not large enough (Cho et al., 2021), so it may not perform as well when detecting non-uniform DIF. To reduce bias in model parameter estimates, we employ an additional importance sampling step after GVEM converges to find a better approximation of the marginal log-likelihood function than the variational lower bound. This idea has recently been used in Ma et al. (2023) to reduce the estimation bias of GVEM for (single-group) MIRT models.

Recall that we obtained a lower bound of the marginal log-likelihood function (4) by Jensen’s inequality, 

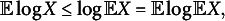

where the last equality holds because 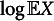 is a constant. To obtain a tighter bound, we hope to find a random variable *Y* such that 

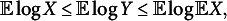

so a natural choice is the empirical mean, i.e., 

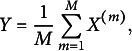

where 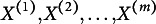 are independent and have the same distribution as *X*. Applying this idea to the marginal log-likelihood function 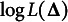 in (4), we can sample from the estimated variational distributions of latent traits and use the importance sampling weighted samples to approximate a tighter variational lower bound than (7). More specifically, after the GVEM algorithm converges, in an additional E-step we draw 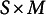 samples 
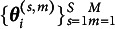
 from estimated 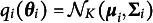 for each person *i*. With these samples, we have the following improved variational lower bound: (8)

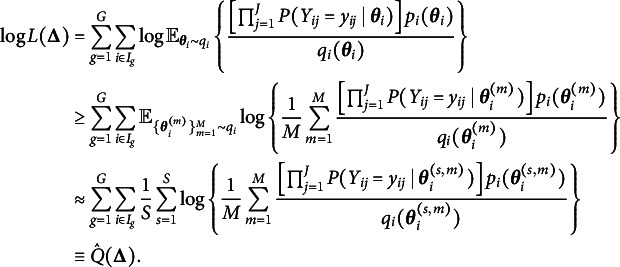

When 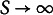 and 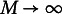 , it can be shown that 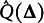 converges in probability to the marginal log-likelihood function 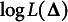 (Burda et al., 2016). In the simulation study we will show that even small values like 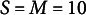 can lead to huge improvement and satisfactory performance. The new objective function to be maximized in the two additional M-steps now becomes (9)

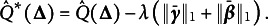

Due to its complexity, there are no closed form updating formulas as in GVEM, so instead we employ gradient-based optimization algorithms.

To ensure the positive definiteness of 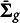 , we conduct Cholesky decomposition 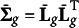 and maximize (10) with respect to 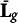 instead of 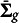 . Furthermore, we let 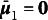 and fix 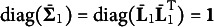 by utilizing the transformation 

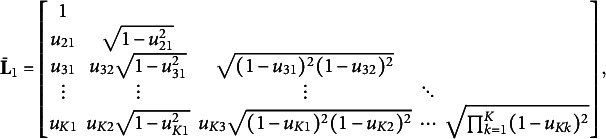

where 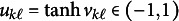 , and 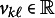 for 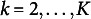 and 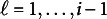 (Lewandowski et al., 2009). Gradients can be computed implicitly by automatic differentiation, such as using the torch package in R (Falbel & Luraschi, 2023).

We apply the Adam optimization algorithm by Kingma and Ba (2014), a popular optimizer in deep learning, to minimize 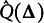 with respect to all the model parameters: 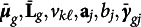 and 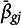 . Adam computes the adaptive learning rate for each parameter based on moving averages of the first and second moments of the gradient, which helps avoid the difficulty in choosing a single proper learning rate for all the parameters. Since 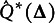 has additional penalty terms that are not differentiable but convex, we apply the proximal gradient method (PGM; Hastie et al., 2015) to 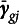 and 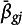 : each entry 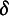 with penalty 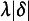 is updated by (11)

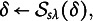

where *s* is the adaptive learning rate used to update 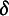 in Adam.

Similar to the regularized GVEM algorithm, we maximize 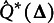 twice in two consecutive M-steps, the first one with a penalty and the second one without a penalty but fixing current zero entries in 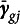 and 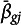 , determined by the first one, at zero. Moreover, we found through simulation that the GVEM algorithm without penalty provides better initial values to the following importance sampling procedure because regularized GVEM does not detect DIF items well and hence gives inaccurate variational distributions of latent traits that importance sampling is based on. Our final algorithm, named “importance-weighted Gaussian variational expectation-maximization-maximization” (IW-GVEMM), is as follows:

1. Obtain initial values: run Steps 1 and 2 of the GVEM algorithm.
2. Conduct Cholesky decomposition and inversely transform parameters: 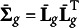 and compute 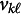 from 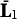 .
3. Draw random samples: draw 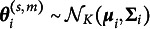 .
4. For each 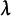 , start from the initial values obtained from Steps 1 to 3:
  - Repeat until convergence with the 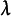 given: in each iteration, update 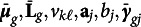 and 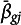 using Adam, and then update 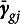 and 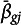 using (11).
  - Repeat until convergence with 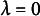 and current zero entries of 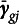 and 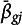 fixed at zero: in each iteration, update 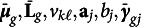 and 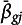 using Adam.

To avoid randomness in the E-step of the importance sampling, we only sample 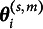 ’s once in Step 3 and then fix them for all values of 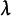 when running Step 4. Since Steps 1 to 3 do not depend on 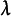 , we only need to run them once for each dataset.

Compared to the Lasso EMM method proposed by Wang et al. (2023) which maximizes the objective function (3) on *K*-dimensional Gaussian quadrature using the Newton-Raphson method, the main advantage of this regularized IW-GVEMM method is that it better handles higher dimensional latent traits because it does not need to compute *K*-dimensional numerical integrals or invert *K*-dimensional matrices, which become very slow and numerically unstable for large *K*.

### 2.4 Information criteria for tuning parameter selection

We use information criteria to find the best tuning parameter 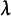 in this study. The marginal log-likelihood 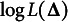 in (4) is difficult to compute because it involves *K*-dimensional numerical integration, but its (approximate) variational lower bounds 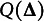 in (7) and 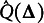 in (9) are by-products of our proposed algorithms. Consequently, we modify the generalized information criterion (GIC; Zhang et al., 2012) 

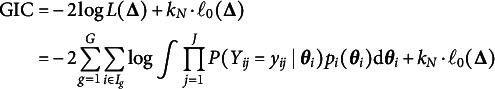

by replacing 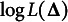 with 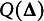 as 

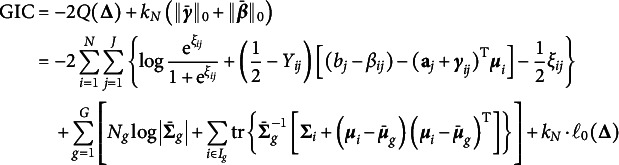

for GVEM, and by replacing 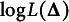 with 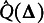 as 

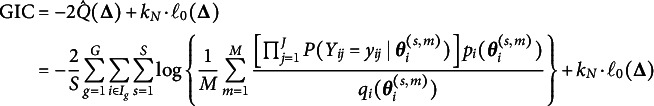

for IW-GVEMM, where 

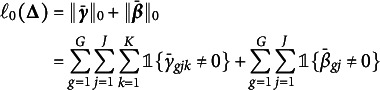

is the number of non-zero DIF parameters and 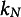 is an increasing function of *N*. In particular, GIC becomes BIC by taking 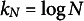 . Our simulation study shows that BIC does not penalize DIF parameters strongly enough and leads to too many false positives under some scenarios. Therefore, we also use 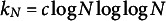 where 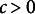 is a prespecified constant that controls the magnitude of penalty, i.e., larger *c* indicates a higher penalty and shrinks more parameters toward zero.

We first apply the GVEM and the IW-GVEMM algorithms with different values of 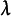 , and after all the estimation is done, we choose the 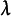 with the lowest information criteria (BIC or GIC). Note that *c* is a constant for model comparison using GIC rather than a model parameter that affects the estimation. Our simulation study shows that 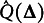 works as a good proxy of 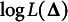 for selecting the best tuning parameter for IW-GVEMM that helps detect DIF.

## 3 Simulation

Two simulation studies are conducted to examine the performance of GVEM and IW-GVEMM algorithms for DIF detection in two-parameter re-MIRT models. Study I focuses on uniform DIF detection and study II focuses on non-uniform DIF detection. In both studies, we set 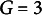 groups, one reference group and two focal groups, where the first focal group has low DIF and the second has high DIF. The latent traits 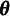 of all the three groups are generated from 

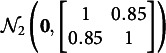

when 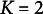 and from 

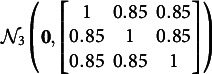

when 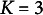 , i.e., there is no impact for the two focal groups. Such high correlations among latent dimensions are not uncommon in practice (Wang et al., 2004), and prior studies showed that high correlations like 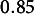 result in more difficulty in model estimation and DIF detection compared to low correlations like 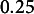 (Cho et al., 2021; Wang et al., 2023). Our pilot study suggests that the correlation has little effect on the running time and the DIF detection accuracy of the proposed approaches. The test length is fixed at 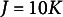 , and each dimension corresponds to 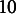 items that load solely on this dimension. In both studies, for the reference group, slopes 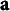 are generated from 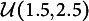 and intercepts 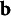 are generated from 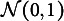 . To evaluate the magnitude of DIF, we compute wABC, the area between expected item score curves for the reference and the focal groups (Edelen et al., 2015). Two factors, sample size *n* of each group ( 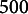 and 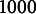 ) and proportion of DIF items ( 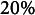 and 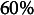 ), are manipulated. For each simulation condition we run 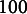 replications. Our convergence criterion is that the absolute difference of every entry 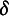 of all the parameters between consecutive iterations (i.e., 
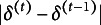
 at the *t*th iteration) is less than 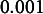 .

For comparison, we also apply the Lasso EMM method proposed by Wang et al. (2023) to the same simulated data. Same as GVEM and IW-GVEMM, EMM also tries to maximize the objective function in (3), but uses Gaussian quadrature rather than Gaussian variational approximation to deal with the integrals. Denser quadrature approximates the integral better and results in higher accuracy and longer computation time. We construct multidimensional Gaussian quadrature using the nested Gauss–Hermite rule (Genz & Keister, 1996) and the sparse combination technique (Heiss & Winschel, 2008). As a result, there are 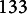 and 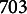 grid points for 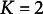 and 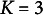 , respectively. More grid points help achieve higher accuracy, but as will be shown later, even with such small numbers of grid points, EMM is much slower than our proposed methods. It is worth noting that since latent dimensions are highly correlated with each other, in several replications the estimates of group-level covariance matrices can be nearly singular after the M-steps of EMM, which makes estimation difficult. For such replications, we have to increase the numbers of grid points to 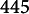 for 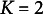 and 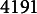 for 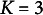 to make EMM work, and they require much longer running time.

Following Cho et al. (2024), we set 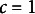 for GIC. We will consider some other possible strategies for choosing *c* in the next section on real data analysis. Within each replication, we estimate the model with eight 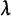 values from 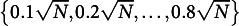 first. If the best model corresponds to 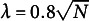 , then we additionally estimate the model with larger tuning parameters, 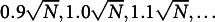 , until the best model does not correspond to the largest 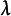 . Under the simplest condition ( 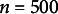 and 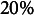 DIF), the mean running times (in seconds) of the first five replications over the eight 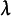 ’s on a MacBook Pro with M3 Max are shown in Table 1, where the numbers of grid points for EMM are 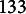 and 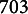 for 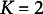 and 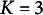 . The two proposed methods show a clear advantage in efficiency compared to the EMM method, especially when the dimension of the latent traits grows. GVEM is faster than IW-GVEMM, but as will be shown later, IW-GVEMM is more accurate. Given the long running time of EMM, we use multiple computers to run the remaining replications, so their running times are not comparable.

**Table 1:** Mean running times (in Seconds) of the first five replications

K	GVEM	IW-GVEMM	EMM
2	5.61	66.98	168.13
3	8.44	85.94	2288.70

### 3.1 Simulation I: Uniform DIF

Under the uniform DIF condition, the slopes for the two focal groups are equal to those for the reference group even for DIF items. Table 2 shows the DIF parameters and the mean wABCs of each condition.

**Table 2:** DIF Parameters in simulation study I

	First half of DIF items			Second half of DIF items		
Group	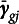	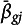	Mean wABC	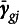	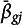	Mean wABC
Low DIF	0	0.5	0.070	0	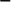 0.5	0.070
High DIF	0	1	0.138	0	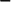 1	0.138

Tables 3 and 4 show the true and the false positive rates of DIF detection across 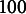 replications, where standard deviations are shown in parentheses. Besides low and high DIF groups, we also show whether items are marked as DIF regardless of low or high DIF group as “Total”. It turns out that importance sampling leads to a huge improvement: true positive rates are much higher, and false positive rates are similar or lower except for BIC with 60% DIF. Moreover, IW-GVEMM has a similar performance to EMM but runs much faster. EMM is better at detecting low DIF under 20% DIF conditions, but this pattern is reversed under 60% DIF. One possible reason is that due to long running time we do not use large numbers of grid points for EMM except for several replications where group-level covariance matrices become singular. With more grid points, EMM is expected to be more accurate, but still it is unlikely that EMM will show very obvious advantages in accuracy over IW-GVEMM. It is worth noting that since IW-GVEMM works on the Cholesky factors of the covariance matrices, it is more robust to high correlations among latent dimensions than EMM. This also helps explain why the performance of EMM is not consistently better than its approximation IW-GVEMM. We found in the pilot study that EMM tends to have better performance than IW-GVEMM when the correlations among the latent traits are lower, which agrees with our explanation here. GIC leads to both lower true positive rates and lower false positive rates in all conditions than BIC, which is expected because GIC penalizes more severely and shrinks more DIF parameters to zero. BIC generally works well for 20% DIF but leads to high false positive rates for 60% DIF; GIC with 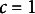 controls false positive rates in all conditions but has difficulty detecting low DIF. In practice, researchers may apply the methods proposed in the next section to find a better *c* for GIC that achieves balance between the true and the false positive rates. Unsurprisingly, the larger sample size leads to higher true positive rates but also slightly higher false positive rates with GIC. Both the proportions of DIF items and the numbers of latent dimensions result in mixed differences.

**Table 3:** Means (standard deviations) of true positive rates across replications of simulation study I

				GVEM		IW-GVEMM		EMM	
K	n	DIF	Group	BIC	GIC	BIC	GIC	BIC	GIC
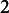	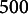		Total	0.715 (0.304)	0.642 (0.314)	0.973 (0.079)	0.882 (0.157)	0.967 (0.092)	0.865 (0.172)
		20%	Low	0.235 (0.321)	0.100 (0.216)	0.340 (0.285)	0.112 (0.182)	0.368 (0.267)	0.132 (0.202)
			High	0.715 (0.304)	0.642 (0.314)	0.973 (0.079)	0.882 (0.157)	0.967 (0.092)	0.865 (0.172)
			Total	0.686 (0.280)	0.517 (0.261)	0.992 (0.028)	0.905 (0.119)	0.983 (0.046)	0.828 (0.166)
		60%	Low	0.169 (0.256)	0.039 (0.124)	0.475 (0.164)	0.123 (0.131)	0.486 (0.193)	0.108 (0.128)
			High	0.686 (0.280)	0.517 (0.261)	0.992 (0.028)	0.905 (0.119)	0.983 (0.046)	0.828 (0.166)
	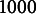		Total	0.887 (0.234)	0.848 (0.266)	0.998 (0.025)	0.990 (0.049)	0.993 (0.043)	0.988 (0.055)
		20%	Low	0.450 (0.364)	0.333 (0.343)	0.733 (0.259)	0.452 (0.260)	0.767 (0.217)	0.472 (0.280)
			High	0.887 (0.234)	0.848 (0.266)	0.998 (0.025)	0.990 (0.049)	0.993 (0.043)	0.988 (0.055)
			Total	0.867 (0.216)	0.811 (0.261)	1.000 (0.000)	0.999 (0.008)	1.000 (0.000)	0.998 (0.014)
		60%	Low	0.449 (0.402)	0.285 (0.330)	0.843 (0.120)	0.583 (0.153)	0.754 (0.152)	0.482 (0.163)
			High	0.867 (0.216)	0.811 (0.261)	1.000 (0.000)	0.999 (0.008)	1.000 (0.000)	0.998 (0.014)
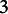	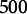		Total	0.725 (0.302)	0.565 (0.333)	0.982 (0.062)	0.877 (0.155)	0.972 (0.082)	0.877 (0.149)
		20%	Low	0.217 (0.284)	0.078 (0.168)	0.358 (0.252)	0.070 (0.130)	0.367 (0.225)	0.127 (0.161)
			High	0.725 (0.302)	0.565 (0.333)	0.982 (0.062)	0.877 (0.155)	0.970 (0.083)	0.877 (0.149)
			Total	0.714 (0.244)	0.516 (0.266)	0.993 (0.018)	0.909 (0.116)	0.964 (0.084)	0.833 (0.174)
		60%	Low	0.151 (0.240)	0.045 (0.127)	0.468 (0.125)	0.104 (0.110)	0.438 (0.171)	0.100 (0.109)
			High	0.713 (0.244)	0.516 (0.266)	0.993 (0.018)	0.909 (0.116)	0.953 (0.112)	0.833 (0.173)
	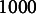		Total	0.857 (0.212)	0.805 (0.273)	1.000 (0.000)	0.995 (0.029)	1.000 (0.000)	0.992 (0.037)
		20%	Low	0.378 (0.363)	0.290 (0.319)	0.735 (0.198)	0.452 (0.231)	0.732 (0.198)	0.452 (0.259)
			High	0.857 (0.212)	0.805 (0.273)	1.000 (0.000)	0.995 (0.029)	1.000 (0.000)	0.992 (0.037)
			Total	0.839 (0.219)	0.783 (0.270)	0.999 (0.006)	0.996 (0.015)	0.999 (0.008)	0.995 (0.021)
		60%	Low	0.343 (0.348)	0.202 (0.243)	0.819 (0.092)	0.500 (0.166)	0.671 (0.139)	0.429 (0.131)
			High	0.839 (0.219)	0.783 (0.270)	0.999 (0.006)	0.996 (0.015)	0.999 (0.008)	0.994 (0.022)

**Table 4:** Means (standard deviations) of false positive rates in simulation study I

				GVEM		IW-GVEMM		EMM	
K	n	DIF	Group	BIC	GIC	BIC	GIC	BIC	GIC
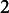	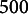		Total	0.064 (0.125)	0.016 (0.048)	0.047 (0.059)	0.002 (0.011)	0.051 (0.063)	0.002 (0.012)
		20%	Low	0.036 (0.082)	0.007 (0.026)	0.024 (0.037)	0.001 (0.009)	0.026 (0.042)	0.001 (0.009)
			High	0.036 (0.085)	0.009 (0.032)	0.024 (0.044)	0.001 (0.006)	0.025 (0.044)	0.001 (0.009)
			Total	0.041 (0.117)	0.004 (0.028)	0.106 (0.104)	0.005 (0.025)	0.164 (0.189)	0.014 (0.061)
		60%	Low	0.019 (0.065)	0.000 (0.000)	0.064 (0.076)	0.001 (0.013)	0.111 (0.143)	0.004 (0.021)
			High	0.025 (0.077)	0.004 (0.028)	0.045 (0.078)	0.004 (0.021)	0.064 (0.117)	0.010 (0.049)
	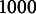		Total	0.087 (0.131)	0.024 (0.052)	0.064 (0.065)	0.009 (0.024)	0.058 (0.069)	0.006 (0.018)
		20%	Low	0.047 (0.091)	0.010 (0.028)	0.029 (0.043)	0.006 (0.021)	0.031 (0.047)	0.004 (0.016)
			High	0.044 (0.079)	0.015 (0.039)	0.035 (0.051)	0.003 (0.014)	0.027 (0.049)	0.001 (0.009)
			Total	0.084 (0.179)	0.015 (0.045)	0.085 (0.108)	0.015 (0.041)	0.141 (0.156)	0.020 (0.049)
		60%	Low	0.052 (0.131)	0.010 (0.038)	0.039 (0.073)	0.005 (0.025)	0.101 (0.150)	0.013 (0.042)
			High	0.050 (0.126)	0.005 (0.025)	0.049 (0.087)	0.010 (0.034)	0.045 (0.082)	0.007 (0.030)
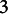	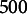		Total	0.055 (0.085)	0.013 (0.038)	0.068 (0.071)	0.003 (0.011)	0.056 (0.056)	0.005 (0.016)
		20%	Low	0.023 (0.045)	0.005 (0.023)	0.030 (0.045)	0.001 (0.006)	0.033 (0.044)	0.003 (0.012)
			High	0.035 (0.058)	0.010 (0.024)	0.040 (0.048)	0.002 (0.009)	0.025 (0.036)	0.002 (0.009)
			Total	0.036 (0.089)	0.006 (0.024)	0.123 (0.106)	0.008 (0.025)	0.258 (0.260)	0.024 (0.060)
		60%	Low	0.022 (0.058)	0.003 (0.016)	0.071 (0.075)	0.006 (0.021)	0.154 (0.178)	0.005 (0.020)
			High	0.020 (0.059)	0.003 (0.016)	0.060 (0.079)	0.003 (0.014)	0.133 (0.227)	0.019 (0.058)
	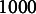		Total	0.032 (0.073)	0.011 (0.030)	0.047 (0.047)	0.010 (0.020)	0.056 (0.080)	0.003 (0.011)
		20%	Low	0.019 (0.053)	0.005 (0.021)	0.025 (0.031)	0.005 (0.013)	0.028 (0.042)	0.001 (0.006)
			High	0.016 (0.034)	0.005 (0.015)	0.023 (0.029)	0.005 (0.015)	0.030 (0.052)	0.002 (0.009)
			Total	0.059 (0.155)	0.011 (0.039)	0.108 (0.090)	0.018 (0.042)	0.180 (0.209)	0.027 (0.053)
		60%	Low	0.034 (0.112)	0.004 (0.022)	0.055 (0.073)	0.011 (0.031)	0.150 (0.210)	0.021 (0.046)
			High	0.034 (0.098)	0.007 (0.023)	0.058 (0.065)	0.007 (0.023)	0.038 (0.059)	0.008 (0.028)

### 3.2 Simulation II: Non-uniform DIF

In the second simulation study, there are DIF effects on both intercepts and slopes, which are shown in Table 5. The true and false positive rates of DIF detection across replications are shown in Tables 6 and 7. All three algorithms perform worse due to the more complex model setting, but the general patterns are largely similar to the uniform DIF simulation study: IW-GVEMM and EMM perform similarly, and both have better performance than GVEM, GIC penalizes more than BIC, and true positive rates increase with larger sample sizes. Besides, although IW-GVEMM and EMM are still good at detecting high DIF, DIF is mostly detected on the intercept 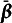 rather than the slope 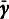 . It is less of a problem in practice because DIF in slopes usually comes with DIF in intercepts.

**Table 5:** DIF parameters in simulation study II

	First half of DIF items			Second half of DIF items		
Group	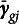	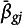	Mean wABC	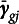	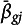	Mean wABC
Low DIF	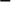 0.4	0.5	0.079	0.4	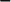 0.5	0.065
High DIF	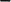 0.8	1	0.170	0.8	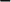 1	0.117

**Table 6:** Means (standard deviations) of true positive rates in simulation study II

				GVEM		IW-GVEMM		EMM	
K	n	DIF	Group	BIC	GIC	BIC	GIC	BIC	GIC
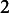	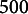		Total	0.683 (0.288)	0.603 (0.291)	0.913 (0.148)	0.752 (0.218)	0.905 (0.150)	0.743 (0.202)
		20%	Low	0.145 (0.264)	0.062 (0.160)	0.380 (0.285)	0.105 (0.164)	0.377 (0.245)	0.117 (0.168)
			High	0.683 (0.288)	0.603 (0.291)	0.913 (0.148)	0.752 (0.218)	0.902 (0.150)	0.743 (0.202)
			Total	0.658 (0.215)	0.521 (0.206)	0.940 (0.073)	0.760 (0.132)	0.928 (0.088)	0.680 (0.171)
		60%	Low	0.128 (0.239)	0.037 (0.129)	0.491 (0.165)	0.102 (0.132)	0.469 (0.184)	0.089 (0.138)
			High	0.657 (0.214)	0.518 (0.203)	0.939 (0.073)	0.760 (0.132)	0.925 (0.088)	0.680 (0.171)
	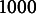		Total	0.800 (0.241)	0.755 (0.256)	0.975 (0.083)	0.928 (0.130)	0.978 (0.080)	0.935 (0.121)
		20%	Low	0.325 (0.392)	0.198 (0.332)	0.678 (0.226)	0.410 (0.274)	0.702 (0.218)	0.447 (0.267)
			High	0.800 (0.241)	0.755 (0.256)	0.975 (0.083)	0.928 (0.130)	0.978 (0.080)	0.935 (0.121)
			Total	0.756 (0.227)	0.686 (0.215)	0.980 (0.049)	0.923 (0.096)	0.978 (0.054)	0.923 (0.096)
		60%	Low	0.312 (0.389)	0.113 (0.234)	0.759 (0.142)	0.409 (0.163)	0.735 (0.159)	0.432 (0.185)
			High	0.756 (0.227)	0.686 (0.215)	0.979 (0.049)	0.923 (0.096)	0.978 (0.058)	0.923 (0.096)
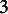	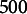		Total	0.732 (0.237)	0.603 (0.259)	0.947 (0.097)	0.760 (0.182)	0.932 (0.106)	0.742 (0.186)
		20%	Low	0.195 (0.304)	0.067 (0.177)	0.385 (0.244)	0.110 (0.181)	0.368 (0.214)	0.122 (0.164)
			High	0.730 (0.235)	0.602 (0.257)	0.938 (0.102)	0.758 (0.181)	0.927 (0.109)	0.740 (0.184)
			Total	0.649 (0.204)	0.517 (0.207)	0.939 (0.059)	0.746 (0.134)	0.911 (0.100)	0.674 (0.150)
		60%	Low	0.111 (0.213)	0.020 (0.079)	0.482 (0.118)	0.093 (0.123)	0.431 (0.172)	0.081 (0.105)
			High	0.649 (0.203)	0.516 (0.206)	0.937 (0.059)	0.746 (0.134)	0.898 (0.107)	0.673 (0.151)
	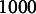		Total	0.760 (0.244)	0.732 (0.246)	0.982 (0.052)	0.932 (0.106)	0.968 (0.070)	0.917 (0.112)
		20%	Low	0.307 (0.392)	0.162 (0.279)	0.702 (0.237)	0.378 (0.251)	0.708 (0.204)	0.390 (0.237)
			High	0.760 (0.244)	0.732 (0.246)	0.982 (0.052)	0.932 (0.106)	0.967 (0.071)	0.917 (0.112)
			Total	0.746 (0.201)	0.668 (0.206)	0.979 (0.034)	0.935 (0.068)	0.976 (0.054)	0.918 (0.106)
		60%	Low	0.229 (0.341)	0.072 (0.156)	0.764 (0.109)	0.443 (0.163)	0.721 (0.145)	0.431 (0.157)
			High	0.746 (0.201)	0.668 (0.205)	0.978 (0.035)	0.935 (0.068)	0.973 (0.055)	0.918 (0.106)

**Table 7:** Means (standard deviations) of false positive rates in simulation study II

				GVEM		IW-GVEMM		EMM	
K	n	DIF	Group	BIC	GIC	BIC	GIC	BIC	GIC
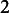	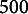		Total	0.031 (0.080)	0.008 (0.035)	0.056 (0.068)	0.002 (0.011)	0.060 (0.070)	0.002 (0.011)
		20%	Low	0.013 (0.041)	0.004 (0.026)	0.025 (0.042)	0.000 (0.000)	0.031 (0.047)	0.001 (0.006)
			High	0.018 (0.050)	0.004 (0.016)	0.031 (0.045)	0.002 (0.011)	0.030 (0.048)	0.001 (0.009)
			Total	0.041 (0.122)	0.007 (0.053)	0.123 (0.112)	0.002 (0.018)	0.145 (0.158)	0.005 (0.025)
		60%	Low	0.021 (0.073)	0.004 (0.021)	0.061 (0.090)	0.001 (0.013)	0.086 (0.120)	0.001 (0.013)
			High	0.026 (0.098)	0.004 (0.038)	0.064 (0.086)	0.001 (0.013)	0.066 (0.110)	0.004 (0.021)
	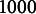		Total	0.071 (0.160)	0.016 (0.064)	0.065 (0.077)	0.010 (0.026)	0.058 (0.070)	0.007 (0.020)
		20%	Low	0.042 (0.116)	0.007 (0.031)	0.035 (0.058)	0.004 (0.018)	0.035 (0.056)	0.004 (0.016)
			High	0.039 (0.093)	0.009 (0.040)	0.031 (0.044)	0.006 (0.020)	0.023 (0.040)	0.003 (0.014)
			Total	0.106 (0.247)	0.009 (0.041)	0.136 (0.134)	0.010 (0.034)	0.151 (0.185)	0.013 (0.038)
		60%	Low	0.071 (0.176)	0.002 (0.025)	0.065 (0.098)	0.002 (0.018)	0.110 (0.159)	0.006 (0.027)
			High	0.060 (0.152)	0.006 (0.033)	0.078 (0.088)	0.007 (0.030)	0.050 (0.110)	0.006 (0.027)
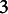	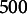		Total	0.054 (0.129)	0.007 (0.028)	0.063 (0.061)	0.005 (0.014)	0.064 (0.075)	0.005 (0.018)
		20%	Low	0.031 (0.084)	0.003 (0.017)	0.035 (0.043)	0.003 (0.012)	0.032 (0.066)	0.001 (0.006)
			High	0.032 (0.087)	0.005 (0.018)	0.031 (0.042)	0.002 (0.009)	0.032 (0.043)	0.004 (0.017)
			Total	0.032 (0.118)	0.003 (0.019)	0.135 (0.106)	0.010 (0.030)	0.198 (0.216)	0.018 (0.045)
		60%	Low	0.017 (0.080)	0.002 (0.017)	0.060 (0.069)	0.003 (0.016)	0.129 (0.165)	0.005 (0.023)
			High	0.019 (0.070)	0.001 (0.008)	0.078 (0.077)	0.007 (0.023)	0.087 (0.160)	0.013 (0.036)
	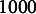		Total	0.062 (0.131)	0.011 (0.045)	0.065 (0.064)	0.004 (0.015)	0.057 (0.057)	0.005 (0.014)
		20%	Low	0.028 (0.063)	0.006 (0.027)	0.035 (0.041)	0.003 (0.012)	0.031 (0.036)	0.002 (0.010)
			High	0.037 (0.084)	0.006 (0.026)	0.032 (0.041)	0.002 (0.010)	0.028 (0.041)	0.003 (0.011)
			Total	0.072 (0.180)	0.008 (0.032)	0.153 (0.118)	0.023 (0.042)	0.259 (0.234)	0.047 (0.073)
		60%	Low	0.051 (0.142)	0.004 (0.022)	0.073 (0.077)	0.010 (0.030)	0.192 (0.209)	0.037 (0.067)
			High	0.048 (0.137)	0.004 (0.022)	0.087 (0.085)	0.013 (0.033)	0.094 (0.147)	0.011 (0.033)

## 4 Real data analysis

To demonstrate the feasibility of the IW-GVEMM algorithm for detecting DIF in real data, we apply it to a dataset from the Patient-Reported Outcomes Measurement Information System (PROMIS) depression and anxiety subscales, which includes responses to 21 items of 5219 cancer patients. The two subscales measure depressive (10 items) and anxiety (11 items) symptoms, respectively, and item content can be found in Table 11 of Wang et al. (2023). Teresi et al. (2016a, 2016b) used this dataset to study DIF on race, a categorical variable with four levels, and we also focus on detecting race DIF here. The reference group is “Non-Hispanic White” (sample size 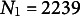 ), and the three focal groups are “Non-Hispanic Black” ( 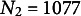 ), “Hispanic” ( 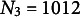 ) and “Non-Hispanic Asians/Pacific Islanders” ( 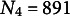 ). All the 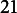 items have ordered categorical responses: “ 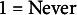 ”, “ 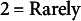 ”, “ 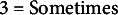 ”, “ 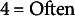 ” and “ 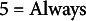 ”, and the proportions that “Never” is chosen fall between 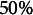 – 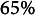 in most items. Therefore, similar to Bauer et al. (2020), we create dichotomous item responses by collapsing all categories except “Never” (i.e., “Rarely”, “Sometimes”, “Often”, and “Always”) to “Yes”, indicating that the patient exhibits this symptom.

Teresi et al. (2016a, 2016b) applied two approaches to detect DIF items. Their first method is the Wald test, which is an iterative method using backward elimination. Initially all the items are assumed to have no DIF and hence work as anchor items. For each anchor item, an IRT model is fit with the constraint that all the current anchor items but this one have the same item parameters across all groups. Then a Wald test is conducted to determine whether the item parameters of this item have significant differences across groups. If so, this item is marked as having DIF and eliminated from the set of anchor items. This procedure is run repeatedly until the set of anchor items stabilizes. Their second method is the ordinal logistic regression, where for each item they regress the response on the group, the latent trait, and their interaction term. An item is marked as having DIF if the group effect or the interaction effect is significantly different from zero. For both methods, they did not seem to model impact but instead assumed a common latent trait distribution for all groups.

**Figure 1:**
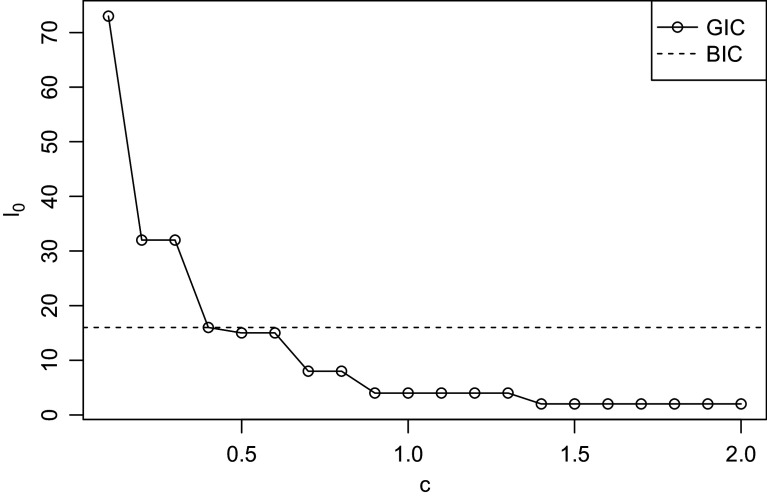
Relationship between number of non-zero DIF parameters and *c* of GIC in PROMIS data.

Before discussing our empirical findings, we propose two possible ways for finding the best constant *c* in GIC for model selection. Figure 1 shows the relationship between 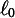 , the number of non-zero DIF parameters of the model with the lowest GIC, against *c*. Figure 1 looks similar to scree plots in principal component analysis, and it suggests that the models chosen by BIC, GIC with 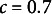 , and GIC with 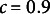 correspond to “elbows” of the plot. Or we may choose *c* by focusing on predictive accuracy as a model fit index. For group *g* and item *j*, we compute the predicted proportion of respondents that choose “Yes” according to the estimated impact and item parameters: 

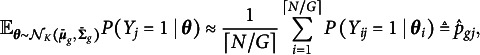

where the expectation is approximated using Monte Carlo integration to accommodate high-dimensional settings, 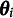 ’s are independently sampled from 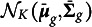 and 
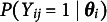
 is defined in (1). Then, we define RMSE as the root mean square error between predicted and observed proportions: 

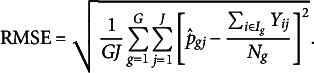

The RMSEs corresponding to GIC with 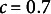 and 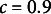 are 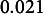 and 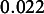 , respectively, suggesting that they have similar model fit. Note that the RMSEs reported here do not serve the purpose of cross-validation because they tend to be smaller for smaller *c* (i.e., more complex models). This also provides support for our default choice of 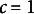 in the simulation study, but still we recommend trying different values of *c* and comparing their results.

Table 8 shows the DIF detection results. DIF is marked by “ 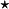 ” for the Wald test and the logistic regression, and these results were obtained directly from Teresi et al. (2016a, 2016b). Since IW-GVEMM found no non-uniform DIF, non-zero estimates of DIF intercept parameters ( 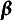 ) are shown instead of “ 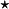 ” in Table 8. DIF items detected by the three approaches do not agree with each other. In particular, regardless of the information criteria, IW-GVEMM finds much fewer DIF items for the “Non-Hispanic Asians/Pacific Islanders” group. This striking difference may be attributed to the fact that Teresi et al. (2016a, 2016b) did not consider impact, i.e., the differences among groups’ population distributions were not considered. As shown in Table 9, the three focal groups all have higher mean anxiety and depression levels than the reference group, so ignoring this difference will inevitably bias DIF detection. Another possible reason is that ordinal responses are collapsed into binary to use our proposed method, whereas Teresi et al. (2016a, 2016b) used the original ordinal responses for DIF analysis. If DIF is absent between “Never” and “Yes” but is present among the four positive responses that are collapsed into “Yes”, then only their approaches are able to detect DIF. As a result, extending our proposed methods to ordinal responses would be an important and useful future direction.

**Table 8:** DIF detection results of PROMIS anxiety and depression scales

	Wald test			Logistic regression			IW-GVEMM (BIC)			IW-GVEMM (GIC, 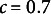 )			IW-GVEMM (GIC, 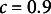 )		
Item	Black	Hisp.	NHAPI	Black	Hisp.	NHAPI	Black	Hisp.	NHAPI	Black	Hisp.	NHAPI	Black	Hisp.	NHAPI
1	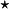	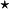	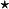	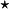	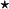	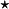									
2			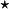	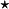	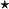	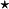	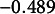								
3			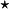	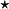	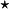	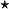									
4					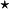	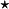		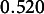							
5	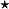		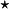	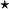	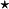	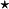		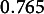	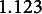		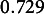	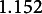			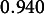
6					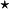	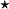									
7		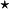	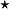		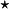	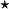		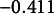			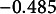				
8		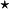	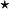		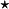	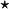		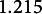			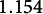			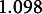	
9	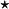	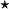	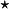	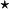	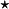	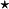	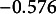		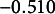	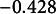					
10	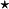	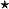	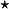	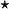	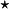	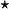		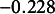							
11			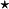		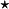	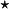		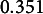							
12	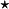	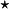	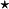	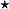	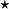	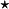	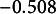	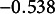			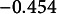				
13		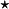		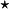		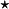	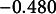								
14	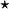	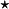		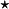		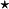									
15					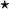	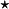									
16						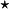									
17	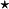					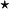			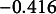						
18			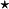	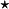	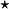	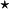		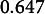			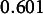			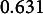	
19			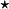	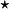	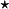	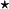									
20			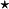	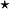		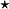		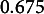			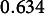			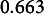	
21						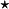									

Reference group: Non-Hispanic White

*Note:* For the Wald test and the logistic regression, “ 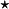 ” indicates DIF detected. IW-GVEMM detected no non-uniform DIF, and non-empty cells display the estimates of DIF intercept parameters 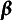 .

**Table 9:** Estimated mean and covariance matrix (impact) of PROMIS anxiety and depression scales using IW-GVEMM

Information criterion	Parameter	White	Black	Hisp.	NHAPI
BIC	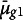	0	0.054	0.216	0.084
	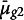	0	0.121	0.265	0.161
	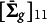	1	1.002	0.962	1.033
	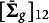	0.941	0.975	0.898	1.010
	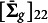	1	1.048	0.957	1.078
GIC, 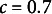	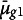	0	0.048	0.222	0.079
	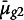	0	0.111	0.271	0.159
	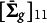	1	1.000	0.956	1.037
	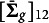	0.941	0.979	0.895	1.012
	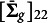	1	1.058	0.956	1.078
GIC, 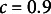	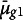	0	0.044	0.227	0.081
	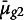	0	0.112	0.267	0.159
	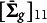	1	0.999	0.966	1.036
	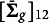	0.942	0.977	0.901	1.011
	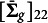	1	1.056	0.959	1.079

## 5 Discussion

This study demonstrates the feasibility of applying regularized IW-GVEMM to detect DIF within the re-MIRT framework. Because all model parameters can be updated in closed forms in the M-step of the GVEM algorithm, it is computationally more efficient than the traditional EM algorithm. However, it may have unsatisfactory performance under non-uniform DIF conditions, which is likely due to the fact that GVEM generates a relatively large bias in discrimination parameters. Such an issue is common in variational estimation for various statistical models (Bishop & Nasrabadi, 2006). As a remedy, we further adopt the importance weighted variational technique (Ma et al., 2023), which gives a tighter variational lower bound of the marginal log-likelihood function. Simulation shows that importance sampling greatly improves the accuracy of estimation, although this additional step is slower due to gradient-based numerical optimization. Information criteria help determine the best tuning parameter 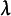 for DIF detection. BIC often works well, but it can lead to inflated false positive rates under some conditions. GIC provides more flexible control over the degree of penalization, but it involves another parameter *c* that may be hard to determine in practice.

This study has certain limitations that suggest potential directions for future research. First, following Wang et al. (2023), although our proposed approach allows and estimates impact, we let all the groups have the same latent trait distribution in the simulation study. Studying the performance of the proposed and other existing methods when impact exists and is large will provide useful guidance for users. Second, we proposed ways to find good values for *c* for GIC, but did not extensively study their performance using simulation because it is beyond the focus of this study. In practice, we may consider cross-validation for model comparison and selection, i.e., split the data into training and test data, fit the models to training data, and then compare their prediction accuracy over test data.

Similar to Wang et al. (2023), we only consider the Lasso or 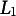 penalty for DIF detection. Due to the inherent bias introduced by Lasso penalty, one additional M-step without penalty is needed. A future direction is to use nonconcave penalties, such as a truncated 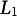 penalty (TLP; Shen et al., 2012), whose idea is to replace (3) by 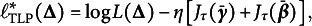 where 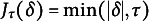 is an elementwise function and 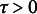 is a tuning parameter. TLP corrects the bias of Lasso by combining adaptive shrinkage with thresholding, so there is no need to run an additional M-step to reduce bias. The optimal tuning parameter may be determined by BIC or GIC as well.

Properly identifying DIF and adjusting for DIF is essential for data harmonization because assuming strict item invariance across groups may be too strict and lead to inaccurate findings. Regularized explanatory MIRT is a flexible modeling framework that simultaneously handles multidimensional traits and potential DIF explained by multiple covariates. It obviates the tedious process of detecting DIF on each item and each covariate one at a time, which is often the case in traditional likelihood-ratio-based DIF detection, and the reliance on modification indices in confirmatory factor analysis. The IW-GVEMM algorithm provides a computationally efficient alternative to the classic EM algorithm, and it can naturally handle high dimensional latent traits. Hence, it has a great potential to serve as a screening tool when analyzing integrated item response data. It is worth noting that we utilize dummy coding when dealing with multiple categorical covariates or one categorical covariate with multiple levels. This requires us to choose one group as a reference and all other groups become focal groups, and the proposed regularized DIF detection method identifies DIF items by comparing each focal group to the reference group. As a result, the proposed method may find different DIF items if a different reference group is chosen. This poses no problem when the goal is to adjust for non-invariance during data harmonization. However, if the goal is to detect DIF, then the selection of a designated reference group needs careful justification because we cannot directly compare two focal groups unless we run the algorithm again where one focal group becomes the new reference group. Hence, future research is needed to develop reference group agnostic DIF detection methods that will pinpoint items behaving differently across pairs of groups without designating a specific reference group.

## Supporting information

Lyu et al. supplementary material Lyu et al. supplementary material

## Data Availability

The code that supports the findings of this study will be available on the project webpage (https://sites.uw.edu/pmetrics/projects/) shortly as we are still working on creating user-friendly R package and Shiny App. The real data will be available upon request.

## Supplementary material

The supplementary material for this article can be found at https://doi.org/10.1017/psy.2024.15.

## References

[r1] Bauer, D. J. (2017). A more general model for testing measurement invariance and differential item functioning. Psychological Methods, 22(3), 507–526. 10.1037/met0000077 27266798 PMC5140785

[r2] Bauer, D. J. , Belzak, W. C. M. , & Cole, V. T. (2020). Simplifying the assessment of measurement invariance over multiple background variables: Using regularized moderated nonlinear factor analysis to detect differential item functioning. Structural Equation Modeling: A Multidisciplinary Journal, 27(1), 43–55. 10.1080/10705511.2019.1642754 33132679 PMC7596881

[r3] Bishop, C. M. , & Nasrabadi, N. M. (2006). Pattern recognition and machine learning, Springer.

[r4] Blei, D. M. , Kucukelbir, A. , & McAuliffe, J. D. (2017). Variational inference: A review for statisticians. Journal of the American statistical Association, 112(518), 859–877.

[r5] Burda, Y. , Grosse, R. , & Salakhutdinov, R. (2016). Importance weighted autoencoders. 10.48550/arXiv.1509.00519

[r6] Carrasco, M. A. , Arias, R. , & Figueroa, M. E. (2017). The multidimensional nature of HIV stigma: evidence from Mozambique. African Journal of AIDS Research, 16(1), 11–18. 10.2989/16085906.2016.1264983 28367746

[r7] Chen, J.-H. , Chen, C.-T. , & Shih, C.-L. (2014). Improving the control of type I error rate in assessing differential item functioning for hierarchical generalized linear model when impact is presented. Applied Psychological Measurement, 38(1), 18–36. 10.1177/0146621613488643

[r8] Chen, Y. , Li, C. , Ouyang, J. , & Xu, G. (2023). DIF statistical inference without knowing anchoring items. Psychometrika, 88(4), 1097–1122. 10.1007/s11336-023-09930-9 37550561 PMC10656337

[r9] Cho, A. E. , Wang, C. , Zhang, X. , & Xu, G. (2021). Gaussian variational estimation for multidimensional item response theory. British Journal of Mathematical and Statistical Psychology, 74(S1), 52–85. 10.1111/bmsp.12219 33064318

[r10] Cho, A. E. , Xiao, J. , Wang, C. , & Xu, G. (2024). Regularized variational estimation for exploratory item factor analysis. Psychometrika, 89(1), 347–375. 10.1007/s11336-022-09874-6 35831697

[r11] Curran, P. J. , & Hussong, A. (2009). Integrative data analysis: The simultaneous analysis of multiple data sets. Psychological Methods, 14(2), 81–100. 10.1037/a0015914 19485623 PMC2777640

[r12] Curran, P. J. , Obeidat, K. , & Losardo, D. (2010). Twelve frequently asked questions about growth curve modeling. Journal of Cognition and Development, 11(2), 121–136. 10.1080/15248371003699969 21743795 PMC3131138

[r13] Debelak, R. , & Strobl, C. (2019). Investigating measurement invariance by means of parameter instability tests for 2PL and 3PL models. Educational and Psychological Measurement, 79(2), 385–398. 10.1177/0013164418777784 30911198 PMC6425091

[r14] Donoho, D. L. , & Johnstone, I. M. (1995). Adapting to unknown smoothness via wavelet shrinkage. Journal of the American Statistical Association, 90(432), 1200–1224. 10.1080/01621459.1995.10476626

[r15] Edelen, M. O. , Stucky, B. , & Chandra, A. (2015). Quantifying ‘problematic’ DIF within an IRT framework: application to a cancer stigma index. Quality of Life Research, 24(1), 95–103. 10.1007/s11136-013-0540-4 24214177

[r16] Falbel, D. , & Luraschi, J. (2023). Torch: Tensors and neural networks with ‘gpu’ acceleration [Computer software manual]. (https://torch.mlverse.org/docs, https://github.com/mlverse/torch).

[r17] Fayers, P. M. (2007). Applying item response theory and computer adaptive testing: the challenges for health outcomes assessment. Quality of Life Research, 16(1), 187–194. 10.1007/s11136-007-9197-1 17417722

[r18] Genz, A. , & Keister, B. (1996). Fully symmetric interpolatory rules for multiple integrals over infinite regions with Gaussian weight. Journal of Computational and Applied Mathematics, 71(2), 299–309. 10.1016/0377-0427(95)00232-4

[r19] Hastie, T. , Tibshirani, R. , & Wainwright, M. (2015, May). Optimization methods. In Statistical learning with sparsity: the lasso and generalizations (pp. 111–154). CRC Press. 10.1201/b18401-7

[r20] Heiss, F. , & Winschel, V. (2008). Likelihood approximation by numerical integration on sparse grids. Journal of Econometrics, 144(1), 62–80. 10.1016/j.jeconom.2007.12.004

[r21] Kingma, D. P. , & Ba, J. (2014)*. Adam: A method for stochastic optimization*. arXiv preprint. 10.48550/arXiv.1412.6980

[r22] Lewandowski, D. , Kurowicka, D. , & Joe, H. (2009). Generating random correlation matrices based on vines and extended onion method. Journal of Multivariate Analysis, 100(9), 1989–2001. 10.1016/j.jmva.2009.04.008

[r23] Ma, C. , Ouyang, J. , Wang, C. , & Xu, G. (2023). A note on improving variational estimation for multidimensional item response theory. Psychometrika, 89, 172–204. 10.1007/s11336-023-09939-0 37979074

[r24] Michel, P. , Baumstarck, K. , Lancon, C. , Ghattas, B. , Loundou, A. , Auquier, P. , & Boyer, L. (2018). Modernizing quality of life assessment: Development of a multidimensional computerized adaptive questionnaire for patients with schizophrenia. Quality of Life Research, 27(4), 1041–1054. 10.1007/s11136-017-1553-1 28343349

[r25] Nance, R. , Delaney, J. , Golin, C. , Wechsberg, W. , Cunningham, C. , Altice, F. , & Springer, S. (2017). Co-calibration of two self-reported measures of adherence to antiretroviral therapy. AIDS Care, 29(4), 464–468. 10.1080/09540121.2016.1263721 27910703 PMC5291764

[r26] Rijmen, F. , & Jeon, M. (2013). Fitting an item response theory model with random item effects across groups by a variational approximation method. Annals of Operations Research, 206(1), 647–662. 10.1007/s10479-012-1181-7

[r27] Shen, X. , Pan, W. , & Zhu, Y. (2012). Likelihood-based selection and sharp parameter estimation. Journal of the American Statistical Association, 107(497), 223–232. 10.1080/01621459.2011.645783 22736876 PMC3378256

[r28] Teresi, J. A. , Ocepek-Welikson, K. , Kleinman, M. , Ramirez, M. , & Kim, G. (2016a). Measurement equivalence of the patient reported outcomes measurement information system® (PROMIS®) anxiety short forms in ethnically diverse groups. Psychological Test and Assessment Modeling, 58(1), 183–219. 28649483 PMC5479355

[r29] Teresi, J. A. , Ocepek-Welikson, K. , Kleinman, M. , Ramirez, M. , & Kim, G. (2016b). Psychometric properties and performance of the patient reported outcomes measurement information system® (PROMIS®) depression short forms in ethnically diverse groups. Psychological Test and Assessment Modeling, 58(1), 141–181. 28553573 PMC5443256

[r30] van de Geer, S. A. (2008). High-dimensional generalized linear models and the lasso. The Annals of Statistics, 36(2), 614–645. 10.1214/009053607000000929

[r31] Wang, C. , Zhu, R. , & Xu, G. (2023). Using lasso and adaptive lasso to identify DIF in multidimensional 2PL models. Multivariate Behavioral Research, 58(2), 387–407. 10.1080/00273171.2021.1985950 35086405

[r32] Wang, W.-C. , Chen, P.-H. , & Cheng, Y.-Y. (2004). Improving measurement precision of test batteries using multidimensional item response models. Psychological Methods, 9(1), 116–136. 10.1037/1082-989X.9.1.116 15053722

[r33] Wilson, M. , De Boeck, P. , & Carstensen, C. H. (2008). Explanatory item response models: A brief introduction. In J. Hartig , E. Klieme , & D. Leutner (Eds.), Assessment of competencies in educational contexts (pp. 91–120). Hogrefe & Huber Publishers.

[r34] Zhang, Y. , Li, R. , & Tsai, C.-L. (2012). Regularization parameter selections via generalized information criterion. Journal of the American Statistical Association, 105(489), 312–323. 10.1198/jasa.2009.tm08013 PMC2911045 20676354

[r35] Zhao, P. , & Yu, B. (2006). On model selection consistency of Lasso. Journal of Machine Learning Research, 7(90), 2541–2563. Retrieved from http://jmlr.org/papers/v7/zhao06a.html

[r36] Zheng, Y. , Chang, C.-H. , & Chang, H.-H. (2013). Content-balancing strategy in bifactor computerized adaptive patient-reported outcome measurement. Quality of Life Research, 22(3), 491–499. 10.1007/s11136-012-0179-6 22538634

