# Supplementary material for: Multi-Group Regularized Gaussian Variational Estimation: Fast Detection of DIF
Source: Psychometrika. 2025 Jan 3;90(1):2–23. doi: 10.1017/psy.2024.15 (PMC12478606; doi:10.1017/psy.2024.15)
Supplement: Lyu et al. supplementary material [file S0033312324000152sup001.zip › GVEM_DIF_supplement.pdf]

Supplementary Material for "Multi-Group Regularized Gaussian Variational Estimation: Fast Detection of DIF"

In this document we list all the closed form updating formulas for the GVEM algorithm. Most of these formulas are multi-group extensions of those in Cho et al. (2021), while L_1-penalized parameters $\bar{\boldsymbol{\gamma}}_{gj}$ and $\bar{\beta}_{gj}$ are updated in a similar way to those in Wang et al. (2023).

In the E-step, the variational distribution of each latent variable $q_i(\boldsymbol{\theta}_i) = \mathcal{N}_K(\boldsymbol{\mu}_i, \boldsymbol{\Sigma}_i)$ is updated by

$$\boldsymbol{\Sigma}_i^{-1} \leftarrow \bar{\boldsymbol{\Sigma}}_g^{-1} + 2\sum_{j=1}^{J} \eta(\xi_{ij})(\boldsymbol{a}_j + \boldsymbol{\gamma}_{ij})(\boldsymbol{a}_j + \boldsymbol{\gamma}_{ij})^\mathsf{T}$$

and

$$\boldsymbol{\mu}_i \leftarrow \boldsymbol{\Sigma}_i \left\{ \sum_{j=1}^{J} \left[\left(Y_{ij} - \frac{1}{2} \right) + 2\eta(\xi_{ij})(b_j - \beta_{ij}) \right] (\boldsymbol{a}_j + \boldsymbol{\gamma}_{ij}) + \bar{\boldsymbol{\Sigma}}_g^{-1} \bar{\boldsymbol{\mu}}_g \right\},$$

where $i \in I_g$. The Gaussian approximation makes it easier to compute the expectation of $Q(\boldsymbol{\Delta})$ with respect to $q_i(\boldsymbol{\theta}_i)$ than to $p(\boldsymbol{\theta}_i \mid \boldsymbol{y}_i)$.

In the M-step, each model parameter in $\boldsymbol{\Delta}$ is updated separately. The details are given below.

- Local variational parameter ξ_{ij} is updated by

$$\xi_{ij} \leftarrow \left[(b_j - \beta_{ij})^2 - 2(b_j - \beta_{ij})(\boldsymbol{a}_j + \boldsymbol{\gamma}_{ij})^\mathsf{T}\boldsymbol{\mu}_i + (\boldsymbol{a}_j + \boldsymbol{\gamma}_{ij})^\mathsf{T}\left(\boldsymbol{\Sigma}_i + \boldsymbol{\mu}_i\boldsymbol{\mu}_i^\mathsf{T}\right)(\boldsymbol{a}_j + \boldsymbol{\gamma}_{ij})\right]^{\frac{1}{2}}.$$

  Although there are two solutions to $\dfrac{\partial Q}{\partial \xi_{ij}} = 0$ that are negatives of each other, we can just take the positive one since $\eta(\xi_{ij}) = \eta(-\xi_{ij})$.

- Latent trait distribution parameters $\bar{\boldsymbol{\mu}}_g$ and $\bar{\boldsymbol{\Sigma}}_g$ are updated by

$$\bar{\boldsymbol{\mu}}_g \leftarrow \frac{1}{N_g}\sum_{i \in I_g} \boldsymbol{\mu}_i$$

  and

$$\bar{\boldsymbol{\Sigma}}_g \leftarrow \frac{1}{N_g}\left[\sum_{i \in I_g} \boldsymbol{\Sigma}_i + (\boldsymbol{\mu}_i - \bar{\boldsymbol{\mu}}_g)(\boldsymbol{\mu}_i - \bar{\boldsymbol{\mu}}_g)^\mathsf{T}\right].$$

- Item discrimination and difficulty parameters $\boldsymbol{a}_j$ and b_j are updated by setting their corresponding first derivatives

$$\frac{\partial Q}{\partial \boldsymbol{a}_j} = \sum_{i=1}^{N}\left(Y_{ij} - \frac{1}{2}\right)\boldsymbol{\mu}_i + 2\eta(\xi_{ij})\left[(b_j - \beta_{ij})\boldsymbol{\mu}_i - \left(\boldsymbol{\Sigma}_i + \boldsymbol{\mu}_i\boldsymbol{\mu}_i^\mathsf{T}\right)(\boldsymbol{a}_j + \boldsymbol{\gamma}_{ij})\right]$$

  and

$$\frac{\partial Q}{\partial b_j} = \sum_{i=1}^{N}\left(\frac{1}{2} - Y_{ij}\right) - 2\eta(\xi_{ij})\left[(b_j - \beta_{ij}) - (\boldsymbol{a}_j + \boldsymbol{\gamma}_{ij})^\mathsf{T}\boldsymbol{\mu}_i\right]$$

  to zero, which results in closed form solutions

$$\begin{aligned}\{\boldsymbol{a}_j\}_{K_j} \leftarrow &\left[\sum_{i=1}^{N} 2\eta(\xi_{ij})\left\{\boldsymbol{\Sigma}_i + \boldsymbol{\mu}_i\boldsymbol{\mu}_i^\mathsf{T}\right\}_{K_j}\right]^{-1} \\ &\times \left\{\sum_{i=1}^{N}\left(Y_{ij} - \frac{1}{2}\right)\boldsymbol{\mu}_i + 2\eta(\xi_{ij})\left[(b_j - \beta_{ij})\boldsymbol{\mu}_i - \left(\boldsymbol{\Sigma}_i + \boldsymbol{\mu}_i\boldsymbol{\mu}_i^\mathsf{T}\right)\boldsymbol{\gamma}_{ij}\right]\right\}_{K_j}\end{aligned}$$

  and

$$b_j \leftarrow \left[\sum_{i=1}^{N} 2\eta(\xi_{ij})\right]^{-1}\left\{\sum_{i=1}^{N}\left(\frac{1}{2} - Y_{ij}\right) + 2\eta(\xi_{ij})\left[\beta_{ij} + (\boldsymbol{a}_j + \boldsymbol{\gamma}_{ij})^\mathsf{T}\boldsymbol{\mu}_i\right]\right\}.$$

Note that $\{\boldsymbol{a}_j\}_{-K_j} = \mathbf{0}$ because item j loads on dimensions in K_j only.

- To update DIF parameters $\bar{\boldsymbol{\gamma}}_{gj}$ and $\bar{\beta}_{gj}$ which are L_1 penalized in Q_j with tuning parameter $\lambda > 0$, we adopt a quadratic approximation approach similar to Wang et al. (2023): the closed form update rule of entry δ with respect to objective function Q is

$$\delta \leftarrow -\frac{\mathcal{S}_\lambda\left(\dfrac{\partial Q}{\partial \delta} - \delta \dfrac{\partial^2 Q}{\partial \delta^2}\right)}{\dfrac{\partial^2 Q}{\partial \delta^2}},$$

where $\mathcal{S}_\lambda(z) = \mathrm{sign}(z)\max(|z| - \lambda, 0)$ is a soft thresholding operator (Donoho & Johnstone, 1995). Since

$$\begin{aligned}
\frac{\partial Q}{\partial \bar{\boldsymbol{\gamma}}_{gj}} &= \sum_{i \in I_g} \left(Y_{ij} - \frac{1}{2}\right) \boldsymbol{\mu}_i + 2\eta(\xi_{ij}) \left[(b_j - \bar{\beta}_{gj})\boldsymbol{\mu}_i - \left(\boldsymbol{\Sigma}_i + \boldsymbol{\mu}_i \boldsymbol{\mu}_i^\intercal\right)(\boldsymbol{a}_j + \bar{\boldsymbol{\gamma}}_{gj})\right], \\
\frac{\partial^2 Q}{\partial \bar{\boldsymbol{\gamma}}_{gj} \partial \bar{\boldsymbol{\gamma}}_{gj}^\intercal} &= -2 \sum_{i \in I_g} \eta(\xi_{ij}) \left(\boldsymbol{\Sigma}_i + \boldsymbol{\mu}_i \boldsymbol{\mu}_i^\intercal\right), \\
\frac{\partial Q}{\partial \bar{\beta}_{gj}} &= \sum_{i \in I_g} \left(Y_{ij} - \frac{1}{2}\right) + 2\eta(\xi_{ij}) \left[(b_j - \bar{\beta}_{gj}) - (\boldsymbol{a}_j + \bar{\boldsymbol{\gamma}}_{gj})^\intercal \boldsymbol{\mu}_i\right], \\
\frac{\partial^2 Q}{\partial \bar{\beta}_{gj}^2} &= -2 \sum_{i \in I_g} \eta(\xi_{ij}),
\end{aligned}$$

we update $\bar{\boldsymbol{\gamma}}_{gjk}$ where $k \in K_j$ and $\bar{\beta}_{gj}$ by

$$\bar{\gamma}_{gjk} \leftarrow \frac{\left[\mathcal{S}_\lambda\left(\sum_{i \in I_g} \left(Y_{ij} - \frac{1}{2}\right) \boldsymbol{\mu}_i + 2\eta(\xi_{ij}) \left[(b_j - \bar{\beta}_{gj})\boldsymbol{\mu}_i - \left(\boldsymbol{\Sigma}_i + \boldsymbol{\mu}_i \boldsymbol{\mu}_i^\intercal\right)\boldsymbol{a}_j\right]\right)\right]_k}{\left[\sum_{i \in I_g} 2\eta(\xi_{ij}) \left(\boldsymbol{\Sigma}_i + \boldsymbol{\mu}_i \boldsymbol{\mu}_i^\intercal\right)\right]_{kk}}$$

and

$$\bar{\beta}_{gj} \leftarrow \frac{\mathcal{S}_\lambda\left(\sum_{i \in I_g} \left(Y_{ij} - \frac{1}{2}\right) + 2\eta(\xi_{ij}) \left[b_j - (\boldsymbol{a}_j + \bar{\boldsymbol{\gamma}}_{gj})^\intercal \boldsymbol{\mu}_i\right]\right)}{\sum_{i \in I_g} 2\eta(\xi_{ij})}$$

for $g = 2, \ldots, G$ while fixing $\bar{\boldsymbol{\gamma}}_{1j} = \mathbf{0}$ and $\bar{\beta}_{1j} = 0$ for the reference group.

When there is no penalty term such that $\lambda = 0$, by directly setting both $\dfrac{\partial Q}{\partial \bar{\boldsymbol{\gamma}}_{gj}}$ and $\dfrac{\partial Q}{\partial \bar{\beta}_{gj}}$ to zero, we obtain their closed form update rules

$$\{\bar{\boldsymbol{\gamma}}_{gj}\}_{K_j} \leftarrow \left[\sum_{i \in I_g} 2\eta(\xi_{ij}) \left\{\boldsymbol{\Sigma}_i + \boldsymbol{\mu}_i \boldsymbol{\mu}_i^{\mathsf{T}}\right\}_{K_j}\right]^{-1} \times \left\{\sum_{i \in I_g} \left(Y_{ij} - \frac{1}{2}\right) \boldsymbol{\mu}_i + 2\eta(\xi_{ij}) \left[(b_j - \bar{\beta}_{gj})\boldsymbol{\mu}_i - \left(\boldsymbol{\Sigma}_i + \boldsymbol{\mu}_i \boldsymbol{\mu}_i^{\mathsf{T}}\right) \boldsymbol{a}_j\right]\right\}_{K_j}$$

and

$$\bar{\beta}_{gj} \leftarrow \left[\sum_{i \in I_g} 2\eta(\xi_{ij})\right]^{-1} \left\{\sum_{i \in I_g} \left(Y_{ij} - \frac{1}{2}\right) + 2\eta(\xi_{ij}) \left[b_j - (\boldsymbol{a}_j + \bar{\boldsymbol{\gamma}}_{gj})^{\mathsf{T}} \boldsymbol{\mu}_i\right]\right\}.$$

- Finally, for model identification, we fix $\bar{\boldsymbol{\mu}}_1 = \mathbf{0}$ and $\operatorname{diag}(\bar{\boldsymbol{\Sigma}}_1) = \mathbf{1}$ by letting

$$\begin{aligned}
b_j &\leftarrow b_j - \boldsymbol{a}_j^{\mathsf{T}} \bar{\boldsymbol{\mu}}_1, \\
\bar{\beta}_{gj} &\leftarrow \bar{\beta}_{gj} + \bar{\boldsymbol{\gamma}}_{gj}^{\mathsf{T}} \bar{\boldsymbol{\mu}}_1, \\
\boldsymbol{\mu}_i &\leftarrow \boldsymbol{\mu}_i - \bar{\boldsymbol{\mu}}_1, \\
\bar{\boldsymbol{\mu}}_g &\leftarrow \bar{\boldsymbol{\mu}}_g - \bar{\boldsymbol{\mu}}_1, \\
\boldsymbol{a}_j &\leftarrow \bar{\boldsymbol{\Lambda}}_1 \boldsymbol{a}_j, \\
\bar{\boldsymbol{\gamma}}_{gj} &\leftarrow \bar{\boldsymbol{\Lambda}}_1 \bar{\boldsymbol{\gamma}}_{gj}, \\
\boldsymbol{\Sigma}_i &\leftarrow \bar{\boldsymbol{\Lambda}}_1^{-\mathsf{T}} \boldsymbol{\Sigma}_i \bar{\boldsymbol{\Lambda}}_1^{-1}, \\
\bar{\boldsymbol{\Sigma}}_g &\leftarrow \bar{\boldsymbol{\Lambda}}_1^{-\mathsf{T}} \bar{\boldsymbol{\Sigma}}_g \bar{\boldsymbol{\Lambda}}_1^{-1}
\end{aligned}$$

for $j = 1, \ldots, J$ and $g = 1, \ldots, G$, where $\bar{\boldsymbol{\Lambda}}_1 = \operatorname{diag}\left(\sqrt{\operatorname{diag}(\bar{\boldsymbol{\Sigma}}_1)}\right)$ is a diagonal matrix whose diagonal elements are the square roots of those of $\bar{\boldsymbol{\Sigma}}_1$.

References

Cho, A. E., Wang, C., Zhang, X., & Xu, G. (2021). Gaussian variational estimation for multidimensional item response theory. *British Journal of Mathematical and Statistical Psychology*, *74*(S1), 52–85. https://doi.org/10.1111/bmsp.12219

Donoho, D. L., & Johnstone, I. M. (1995). Adapting to unknown smoothness via wavelet shrinkage. *Journal of the American Statistical Association*, *90*(432), 1200–1224. https://doi.org/10.1080/01621459.1995.10476626

Wang, C., Zhu, R., & Xu, G. (2023). Using lasso and adaptive lasso to identify DIF in multidimensional 2PL models. *Multivariate Behavioral Research*, *58*(2), 387–407. https://doi.org/10.1080/00273171.2021.1985950
